# Socio‐Demographic Determinants of Mediterranean Diet Adherence: Results of the EU‐National Health Interview Survey (EHIS‐3)

**DOI:** 10.1111/jhn.70023

**Published:** 2025-02-11

**Authors:** Ioanna Kontele, Demosthenes Panagiotakos, Mary Yannakoulia, Tonia Vassilakou

**Affiliations:** ^1^ Department of Public Health Policy University of West Attica Athens Greece; ^2^ Department of Nutrition and Dietetics Harokopio University Athens Greece

**Keywords:** education, employment, food policy, income, marital status, nutrition

## Abstract

**Background:**

Despite the well‐known benefits of the Mediterranean diet (MD), low adherence is observed globally, highlighting the need to investigate the underlying causes of this trend. Large‐scale, periodically repeated surveys, such as the European Health Interview Survey (EHIS), could be useful for the investigation of the factors that influence adherence to healthy dietary patterns. National EHIS‐3 in Greece was designed to collect data on the consumption of all food groups, making it possible to determine adherence to MD.

**Methodology:**

This study aimed to investigate the socioeconomic factors that influence MD adherence by conducting a secondary data analysis from 7706 participants aged 15–85 years who participated in the 2019 national EHIS. MD adherence was evaluated by MedDietScore. Bivariate comparisons between the score tertiles and socioeconomic groups were performed. Logistic regression analyses were used to estimate the association between MedDietScore tertiles and demographic and socioeconomic factors, using high adherence as the reference category. Finally, cluster analysis was applied to identify the most significant factors in relation to the classification of the participants in MD adherence groups.

**Results:**

Educational level, followed by income status, emerged as the most significant factor associated with adherence to MD. Individuals who have attained only primary education had a 3.80 times higher likelihood of presenting low MD adherence instead of high MD adherence compared to persons with tertiary education. Individuals in the lower income group had 2.53 times higher odds of being in the low MD adherence instead of high MD adherence than individuals in the higher income group. These relationships remained statistically significant after adjusting for confounding factors. The group that most frequently adhered to high MD (53.4%) comprised individuals who had tertiary education, higher income, and were couples without children. Conversely, those with primary or secondary education who are single parents or live in one‐person households and fall into the lower and medium income groups are most likely to have low MD adherence (44.8%).

**Conclusions:**

Public policies to promote MD adherence should consider socioeconomic factors. Integrating questions to evaluate adherence to dietary patterns into EHIS would allow for future intercountry and longitudinal comparisons.

## Summary

- Adherence to the MD pattern was low to moderate across all age groups.
- Individuals who had attained only primary education had higher odds to present low MD adherence compared to individuals with tertiary education.
- Individuals in the lower income group had higher odds of being in the low MD adherence tertile compared to individuals in the higher income group.
- The group with the highest MD adherence was those with tertiary education, higher income and living as couples without children.
- The group that had the highest odds of being low‐adherent to MD were those with primary or secondary education, who live in one‐person households or as lone parents, and who have lower incomes.
- Education and income status were associated with compliance with national food‐based dietary guidelines, as adherence rates for almost every guideline increased as the levels of education and income increased.
- Examination of adherence to eating patterns rather than the consumption of specific food groups could be integrated into EHIS and other large‐scale studies.

## Graphical abstract

Socio‐demographic Determinants of Mediterranean Diet Adherence in Greece.

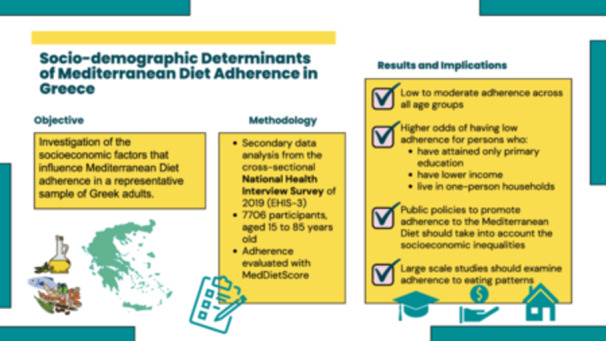

## 1 Introduction

Aiming at the global transformation of food systems, different dietary patterns have been examined regarding their influence on health and the environment [1, 2]. A dietary pattern based on plant‐based foods combined with moderate or limited quantities of animal‐based foods is linked to a reduced environmental impact and is healthier, according to a significant body of research [1]. The traditional Mediterranean diet (MD) is an example of such a pattern, as it is characterized by a large amount of plant foods, such as fruits, vegetables, cereals, legumes and nuts; moderate amounts of fish, poultry, eggs and dairy; and low amounts of red meat, while olive oil is the primary source of fat [3].

Greater adherence to the MD pattern has been associated with a lower risk of mortality and morbidity, especially in cardiovascular diseases, cancer, diabetes and neurodegenerative disorders [4, 5, 6, 7, 8]. Furthermore, over the last few decades, the social and environmental sustainability dimensions of MD patterns have been investigated. Traditional MD provides socio‐cultural, economic and environmental benefits, as biodiversity, local food production, culture and environmental protection are interconnected [9, 10, 11].

Despite the well‐known benefits of MD, a recent systematic review of 57 studies with over 1 million participants worldwide indicated moderate adherence to the MD, with decreasing trends worldwide [12]. In accordance with these findings, a systematic review of studies conducted in Mediterranean countries revealed that adult populations have had low to moderate adherence to MD during the last decade [13]. Studies in Greek populations have shown that less than one‐third of adults have high adherence to traditional Greek MD [14, 15, 16].

Understanding the causes of people's deviation from healthy eating patterns is necessary to design public nutrition policies. Factors related to food availability, climate conditions, tradition and agricultural policies have been proposed as possible contributors to decreased MD adherence [12, 17]. The high cost of certain foods in the MD pattern has also been suggested as a potential factor that emerged during the economic crisis of the last few decades [18, 19]. Moreover, the increased availability and affordability of convenience‐processed foods steer people away from healthy eating habits [20]. The effects of socioeconomic factors on dietary habits have also been investigated. A pooled analysis of consumption data across 12 European countries indicated that participants with higher education levels had better nutritional intake, especially in lower‐income countries [21]. A large study in Italy found that people with higher incomes consume fish, fruits and vegetables more frequently and meat and dairy less frequently than their counterparts with lower incomes [22].

Associations between socioeconomic factors and adherence to specific eating patterns, such as the MD pattern, have also recently been examined. Studies in Italy have indicated that adherence to the MD pattern is determined by sex, age, geographical area, income and educational level [22, 23]. A Portuguese study revealed that younger individuals in lower income categories and with a lower educational level had higher odds of presenting low adherence to MD [24], while a study in Australia found that participants who were married, employed, and had high socioeconomic levels were more likely to have higher adherence levels to MD [25]. Studies in Greece have shown that higher MD adherence scores are associated with higher educational levels [26] and living in regions with lower unemployment rates [27]. However, these studies were based only on regional data and/or specific population groups. A recent systematic review revealed that increasing MD adherence coincided with an increasing Human Developmental Index (which consists of three indicators: life expectancy, years of education and per capita income) in several countries, but countries with Mediterranean‐type ecosystems with lower socioeconomic status showed higher MD adherence [12]. Nevertheless, only a relatively small number of studies have examined the relationship between MD adherence and socioeconomic inequalities in nationally representative samples, while most have reported only a few socioeconomic factors [14, 22, 25, 28, 29].

An effective source of these data could be international multipurpose surveys that are periodically conducted in nationally representative groups. The European Health Interview Survey (EHIS) is conducted every 5 years under a common methodology in all EU countries. Nevertheless, until 2014, only data regarding the consumption of fruits, vegetables and red meat were collected. In 2019, the consumption of legumes and soft drinks was also added to the EHIS protocol, but Greece was the only country that managed to collect data regarding the consumption of 11 food groups, making it possible to determine adherence to an eating pattern of a nationally representative sample. In addition, a large number of socioeconomic characteristics were collected, which allowed for the evaluation of several associations.

Thus, the current secondary data analysis study aimed to evaluate the levels of MD adherence according to the socioeconomic profiles (using a variety of socioeconomic factors) in a nationally representative sample of Greek adults who participated in the third wave of the EHIS, conducted in 2019.

Highlighting the socioeconomic factors that influence adherence to healthy dietary patterns is important to provide evidence for public nutrition policies and interventions that will focus on specific population groups according to their needs. Thus, it is anticipated that these findings will be useful for public health authorities to design more effective nutrition policies. Furthermore, the results of this study are expected to facilitate the addition of more nutrition‐related questions to the EHIS questionnaire, enabling cross‐national and longitudinal comparisons.

## 2 Methods

### 2.1 Study Design

To examine the level of MD adherence in the Greek population and possible correlations with demographic and socioeconomic factors, a secondary analysis of data from the National Health Interview Survey was conducted. The National Health Interview Survey is part of the EHIS, in which all EU member states participate. EHIS is a cross‐sectional country‐level representative study conducted under a common methodology in all participating countries to collect reliable data on health status, healthcare use, health determinants, and socio‐economic and demographic factors. The EHIS targets the population who are at least 15 years old and live in private households; it is conducted every 5 years and was conducted for the first time in 2009 (EHIS‐Wave 1), while EHIS‐Wave 3 took place in all European countries between 2018 and 2020 [30]. Data collection for the EHIS‐3 in Greece took place between October and December 2019.

For the current study, data available in public use files were obtained through the official portal of the Hellenic Statistical Authority (www.statistics.gr), while access to anonymized microdata was obtained upon request from the Statistical Data Dissemination Section of the Statistical Information and Publications Division of the Hellenic Statistical Authority.

### 2.2 Participants

Multistage stratified sampling was applied, with the primary survey unit being the area (one or more building blocks or a small settlement), the secondary unit being the household, and the final unit being the person over 15 years of age (target population). The NUTS2 (level 2 of the Nomenclature of Territorial Units for Statistics) was used for the regions of residence. The final sample is representative of the Greek adult population aged 15 years or over residing in private households [31].

A letter was sent before the study initiation to all the selected households, aiming to inform and also create a context of familiarity, trust, and cooperation with the households. The letter referred to the purpose of the research, the period of time during which the collection was carried out, the preparation of the resulting indicators, the randomness of the selection of the specific household, data confidentiality, the General Regulation for the Protection of Personal Data (GDPR), and compliance with privacy [31].

Overall, 8125 individuals aged 15–85 years participated in the study. For 188 individuals, data were obtained from other members of the household, whereas data for 89 individuals were obtained from a person outside the household. Those 277 participants were excluded from the current analysis. Moreover, 55 participants were excluded due to missing data, and 87 cases were excluded due to outlier data; thus, a total of 7706 subjects were included in the analysis.

### 2.3 Variables and Methods of Measurement

Data were collected through face‐to‐face interviews using an interviewer‐administered non‐electronic version of the EHIS model questionnaire. The questionnaire was completed in one session in each participant's household. The interviewers were either permanent staff of the Hellenic Statistical Authority or externally trained collaborators from the Statistical Interviewers' Register of Hellenic Statistical Authority.

The EHIS model questionnaire consists of four modules: health status, healthcare use, health determinants and socioeconomic background variables. Demographic and socioeconomic characteristics collected included sex, age, region of residence, degree of urbanization, educational attainment level, employment status, income, marital status, household type and household size. The region of residence and degree of urbanization were defined by the Hellenic Statistical Authority. Four regions were recognized: Attica (the prefecture with the largest population in the country, where the capital city Athens is located), Crete and Aegean Islands, Northern Greece and Central Greece. The degree of urbanization was categorized as urban, semi‐urban and rural. Age was reported by the participants in absolute numbers, whereas for all other variables, participants had to choose the most suitable answer from a predefined list. Educational level was reported according to the 2011 version of the International Standard Classification of Education (ISCED 2011) [32]. The income status categories were pre‐defined by the Hellenic Statistics Authority, following the guidelines of Eurostat's Methodological Manual for EHIS‐3 [31], and consisted of five categories according to net monthly household income. Regarding marital status, participants reported whether they were married (or in a registered relationship), widowed, divorced or never married (or never in a registered relationship). Finally, five options regarding household type were used: couples without children, couples with at least one child aged less than 25 years, couples with all children aged 25 years or more, one‐person household, single parent with at least one child aged less than 25 years, and a single parent with all children aged 25 years or more or other types of household. Data on sociodemographic factors refer to the current situation on the day of data collection.

Data on food intake were collected through an interviewer‐administered food frequency questionnaire and were referred to a typical week. The EHIS model questionnaire included questions regarding the consumption of fruits, vegetables, legumes and soft drinks. The Greek HIS questionnaire also included questions regarding the frequency of consumption of non‐refined cereals, full‐fat dairy products, red meat, poultry, fish/seafood, potatoes and olive oil. For all food groups, the possible answers were ‘once or more a day’, ‘4–6 times a week’, ‘1–3 times a week’, ‘less than once a week’, ‘never’. For answers ‘once or more a day’, participants also provided data regarding the number of daily portions. Interviewers used written information regarding the type of food within each food group and the portion sizes, as well as pictures of portions as presented in the National Dietary Guidelines for Adults [33].

Alcohol consumption was assessed through a set of questions that included data on the frequency of consumption separately for weekdays (Monday–Thursday) and weekends (Friday–Sunday), while the average number of alcoholic drinks was also recorded separately for each day. The average alcohol servings per day were calculated as the sum of alcoholic drinks on weekdays and weekends divided by 7.

Adherence to the MD pattern was evaluated using MedDietScore [34]. The score is based on the monthly consumption of non‐refined cereals, potatoes, fruits, vegetables, legumes, fish, red meat, poultry and full‐fat dairy products, weekly use of olive oil, and daily consumption of alcohol. A score of 0–5 was assigned to each food group. Specifically, for the intake of food groups compatible with the MD pattern (fruits, vegetables, legumes, non‐refined cereals, fish, olive oil and potatoes), the more frequent the consumption, the higher the score (5 for daily consumption and 0 for never). However, consumption of red meat, poultry and full‐fat dairy products was given a reverse score scale, as these foods are considered incompatible with the MD pattern. Regarding alcohol consumption, a score of 5 was assigned for consumption of less than 300 mL/day, 4 for 600–700 mL/day, 3 for 500–600 mL/day, 2 for 400–500 mL/day, 1 for 300–400 mL/day and a score of 0 for consumption of more than 700 mL/day, as well as for none. The total MedDietScore ranged from 0 to 55, with higher scores indicating higher adherence to the MD pattern. For analysis purposes, tertiles of MedDietScore were used, with the first tertile showing low adherence, the second tertile showing moderate adherence, and the third tertile showing high adherence.

### 2.4 Statistical Methods

For analysis purposes, three education‐level categories were created, namely primary education (participants with no formal education and ISCED 1‐primary school), secondary education (participants with ISCED 2–4: lower and upper secondary school, as well as post‐secondary non‐tertiary education), and tertiary education (participants with ISCED 5 to 8 – 3‐year tertiary education, bachelor's, master's, doctoral or equivalent degrees). Regarding the income status, three roughly equal income groups were created to facilitate statistical analysis: the lower income group (< 570€ net monthly income), the medium income group (570€–784€ net monthly income) and the higher income group (> 784€ net monthly income). Three categories were created for employment status: employed, retired and unemployed. The last category also involved persons who were unable to work due to longstanding health problems, students, persons involved in domestic tasks, and persons on compulsory military or civilian services. Finally, three types of households were used: couples without children, couples with children (even adult children than those living with their parents), and one‐person households that were combined with households of single parents.

Demographic and socioeconomic characteristics of the participants are presented as absolute numbers and percentages. Pearson's correlation coefficient (*r*) and a scatterplot were used to investigate the linear association between age and the MedDietScore. The Pearson chi‐square test was used for bivariate comparisons of MD tertiles among the different demographic and socioeconomic groups. Adherence to specific national dietary guidelines [33] was examined and presented as a percentage of compliance for every group of demographic and socioeconomic factors.

Multinomial logistic regression analyses were performed to estimate the association between MedDietScore tertiles and demographic and socioeconomic factors, using high adherence as the reference category. Analyses were performed for the total samples and separately for males and females. Odds ratios (ORs) and corresponding 95% confidence intervals (CIs) were provided. Furthermore, discriminant classification analysis by the calculation of Wilks' Lambda was applied to explore the discriminating ability of each demographic and socioeconomic factor in relation to the classification of the participants in MD adherence groups. The lower the Wilks' Lambda, the better the discriminating ability of the variable. Significance was evaluated using the F‐test. Finally, a decision tree through the algorithm CHAID (Automatic Chi‐square Interaction Detection) was applied to reveal the most significant paths of MD adherence groups.

The significance level was set at *p* < 0.05. All analyses were performed using IBM SPSS Statistics for Windows, Version 29.0.

## 3 Results

The study population consisted of 7706 individuals aged 15–85 years old, with a mean age of 55.6 years (SD 19.0), while 46.0% were 60 years and older. Fifty‐three percent of the participants were female. Thirty percent lived in Attica, and 62.5% lived in urban areas. Almost a quarter of the participants had attained tertiary education, and 64.4% were unemployed or retired. Thirty‐seven percent reported a net monthly household income of less than 570€.

**Figure 1:**
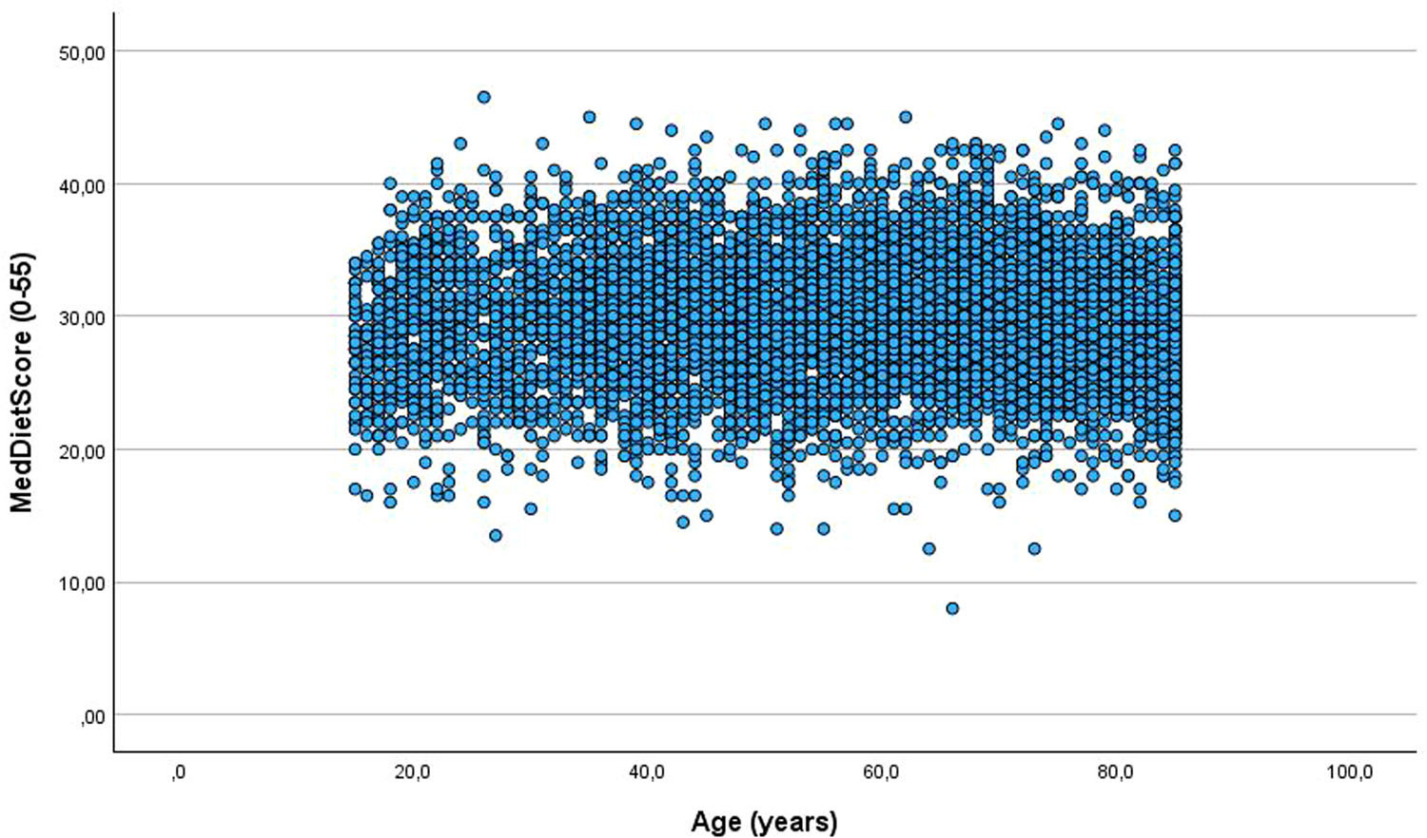
Scatterplot showing the relationship between age and MedDietScore.

Mean MedDietScore of the total sample was 29.8 (SD 4.6), ranging from 8.0 to 46.5. Participants in the first tertile had a mean score of 24.8 (SD 2.4), those in the second tertile 29.8 (SD 1.1), and those in the third tertile 35.0 (SD 2.5). A negative correlation was found between age and MedDietScore, but it was very weak (*r* = −0.023, *p* = 0.041), while the scatterplot (Figure 1) showed no significant differences in MedDietScore across different ages. Thus, there was no significant association between age and MD adherence, and the mean score was low to moderate for all ages.

Table 1 presents the demographic and socioeconomic characteristics of the participants across tertiles of adherence to MD. Education and income status were associated with MD adherence, as 43.9% of the participants with primary education and 41.5% of the low‐income group fell into the low MD adherence category, while 45.1% of the tertiary‐educated participants and 41.6% of the high‐income participants fell into the high MD adherence category. Moreover, a relatively higher percentage of unemployed persons were categorized in the low‐adherence group than in the employed group. Finally, almost 4 out of 10 participants who were widowed, divorced or lived in one‐person households fell into the low‐adherence category, significantly more than the participants who were married or lived with partners.

**Table 1:** Demographic and socioeconomic characteristics of the participants according to levels of adherence to MD.

		Adherence to MD (MedDietScore tertiles)			
	All (*n*)	1st tertile (low)	2nd tertile (moderate)	3rd tertile (high)	*p* value
		*n* (%)	*n* (%)	*n* (%)	
Total	7706	2659 (34.5)	2536 (32.9)	2511 (32.6)	
Sex					
Males	3610	1157 (32.0)	1104 (30.6)	1349 (37.4)	< 0.001
Females	4096	1502 (36.7)	1432 (35.0)	1162 (28.4)	
Region of residence					
Attica	2314	690 (29.8)	709 (30.6)	915 (39.5)	< 0.001
Aegean Islands and Crete	838	255 (30.4)	299 (35.7)	284 (33.9)	
North Greece	2506	959 (38.3)	839 (33.5)	708 (28.3)	
Central Greece	2048	755 (36.9)	689 (33.6)	604 (29.5)	
Degree of urbanization					
Urban areas	5827	1942 (33.3)	1881 (32.3)	2004 (34.4)	< 0.001
Rural areas	1879	717 (38.2)	655 (34.9)	507 (27.0)	
Education level					
Primary education	2225	976 (43.9)	739 (33.2)	510 (22.9)	< 0.001
Secondary education	3605	1258 (34.9)	1192 (33.1)	1155 (32.0)	
Tertiary education	1876	425 (22.7)	605 (32.2)	846 (45.1)	
Income status					
Lower income	2844	1181 (41.5)	937 (32.9)	726 (25.5)	< 0.001
Medium income	2103	741 (35.2)	725 (34.5)	637 (30.3)	
Higher income	2759	737 (26.7)	874 (31.7)	1148 (41.6)	
Employment status					
Employed	2740	814 (29.7)	866 (31.6)	1060 (38.7)	< 0.001
Unemployed	2161	825 (38.2)	760 (35.2)	576 (26.7)	
Retired	2805	1020 (36.4)	910 (32.4)	875 (31.2)	
Marital status					
Married or in a registered partnership	3998	1232 (30.8)	1356 (33.9)	1410 (35.3)	< 0.001
Never married	1830	622 (34.0)	611 (33.4)	597 (32.6)	
Widowed or divorced	1878	805 (42.9)	569 (30.3)	504 (26.8)	
Household type					
Couple with children	2320	754 (32.5)	773 (33.3)	793 (34.2)	< 0.001
Couple without any children	2360	720 (30.5)	796 (33.7)	844 (35.8)	
One‐person household or lone parent	3026	1185 (39.2)	967 (32.0)	874 (28.9)	

Abbreviation: MD, Mediterranean diet.

Moreover, significantly more females than males were categorized as low‐MD adherents. It should be noted that further analysis indicated that this result was due to the low score of females in the alcohol component of the MedDietScore, while they scored higher than males in other components of the scale. To further explore the difference in MD adherence between males and females, it was decided to perform regression analyses for the total sample and separately for males and females. A comparison of dietary patterns between males and females was deemed important in the current analysis as it is known that dietary habits are influenced by sex‐specific characteristics, including sociocultural factors that have different effects on males and females.

Multinomial logistic regression analysis for the overall sample (Table 2) and separately for males (Table 3) and females (Table 4) indicated that the odds of being in the lowest MD tertile increased as the education level and income status decreased. Individuals who have attained only primary education seem to have a 3.8 times higher likelihood of presenting low MD adherence instead of high MD adherence compared to persons with tertiary education. This relationship remained statistically significant in both males and females, even after adjusting for region, degree of urbanization, income, employment status, marital status and household type. Individuals in the lower income group had 2.53 times higher odds of being in the low MD adherence instead of high MD adherence than individuals in the higher income group. Being widowed or divorced was also associated with higher odds of being in the low MD adherence group. This was found for both males and females, but it was not a significant finding in the sex‐specific adjusted models. Accordingly, households of one‐person or lone parents had higher odds of being in the low adherence category, but this finding did not remain significant in the adjusted models. Being unemployed was associated with low MD adherence; however, this result was not significant for females and in the male‐adjusted model. Regarding the different regions, people living in Attica, Aegean Island and Crete had lower odds of being in the low MD adherence tertile than people from other regions.

**Table 2:** Multinomial logistic regression analysis for MD adherence in the overall population by considering all the socioeconomic and demographic variables alone (non‐adjusted ORs) or together (adjusted ORs).

	Low adherence vs. high adherence				Moderate adherence vs. high adherence		
	Non‐adjusted		Adjusted	Wald	Non‐adjusted	Adjusted	Wald
	OR (95% CI)		OR (95% CI)		OR (95% CI)	OR (95% CI)	
Region of residence							
Attica	**0.60 (0.52–0.69)**		**0.76 (0.64–0.89)**	11.11	**0.67 (0.58–0.78)**	**0.79 (0.67–0.93)**	7.94
Aegean Islands and Crete	**0.71 (0.58–0.87)**		**0.72 (0.58–0.88)**	9.78	0.92 (0.75–1.12)	0.93 (0.76–1.13)	0.50
North Greece	1.08 (0.93–1.25)		1.08 (0.93–1.26)	1.22	1.03 (0.89–1.20)	1.05 (0.90–1.22)	0.39
Central Greece	1 (ref)		1 (ref)		1 (ref)	1 (ref)	
Degree of urbanization							
Urban areas	**0.68 (0.60–0.78)**		1.01 (0.87–1.17)	0.02	**0.72 (0.63–0.82)**	0.91 (0.79–1.06)	1.21
Rural areas	1 (ref)		1 (ref)		1 (ref)	1 (ref)	
Education level							
Primary education	**3.80 (3.25–4.46)**		**2.84 (2.33–3.46)**	109.27	**2.02 (1.73–2.36)**	**1.62 (1.34–1.97)**	24.99
Secondary education	**2.16 (1.88–2.49)**		**1.79 (1.53–2.08)**	56.44	**1.44 (1.26–1.64)**	**1.22 (1.05–1.40)**	7.52
Tertiary education	1 (ref)		1 (ref)		1 (ref)	1 (ref)	
Income status							
Lower income	**2.53 (2.22–2.88)**		**1.57 (1.35–1.84)**	34.05	**1.69 (1.48–1.93)**	**1.22 (1.04–1.42)**	6.60
Medium income	**1.81 (1.57–2.08)**		**1.28 (1.10–1.49)**	10.72	**1.49 (1.30–1.71)**	**1.21 (1.04–1.40)**	6.39
Higher income	1 (ref)		1 (ref)		1 (ref)	1 (ref)	
Employment status							
Employed	**0.65 (0.57–0.74)**		1.01 (0.86–1.18)	0.02	**0.78 (0.69–0.89)**	0.93 (0.79–1.08)	0.77
Unemployed	**1.22 (1.06–1.41)**		**1.20 (1.02–1.42)**	4.81	**1.26 (1.10–1.46)**	**1.25 (1.05–1.48)**	6.92
Retired	1 (ref)		1 (ref)		1 (ref)	1 (ref)	
Legal marital status							
Married	**0.54 (0.47–0.62)**		**0.72 (0.56–0.93)**	6.06	**0.85 (0.74–0.98)**	1.14 (0.88–1.48)	1.05
Never married	**0.65 (0.55–0.76)**		**0.79 (0.65–0.97)**	5.16	0.90 (0.76–1.06)	1.04 (0.86–1.27)	0.23
Widowed or divorced	1 (ref)		1 (ref)		1 (ref)	1 (ref)	
Household type							
Couple with children	**0.70 (0.61–0.80)**		1.00 (0.80–1.23)	0.0	0.88 (0.77–1.00)	0.88 (0.71–1.09)	1.27
Couple without any children	**0.62 (0.55–0.71)**		0.80 (0.63–1.03)	2.85	**0.85 (0.74–0.97)**	**0.75 (0.59–0.96)**	4.88
One‐person household or lone parent	1 (ref)		1 (ref)		1 (ref)	1 (ref)	

*Note:* The reference category for MD adherence is high adherence. Estimates with a significance level < 0.05 are highlighted in bold.

Abbreviation: MD, Mediterranean diet.

**Table 3:** Multinomial logistic regression analysis for MD adherence in males by considering all the socioeconomic and demographic variables alone (non‐adjusted ORs) or together (adjusted ORs).

	Low adherence vs. high adherence			Moderate adherence vs. high adherence		
	Non‐adjusted	Adjusted	Wald	Non‐adjusted	Adjusted	Wald
	OR (95% CI)	OR (95% CI)		OR (95% CI)	OR (95% CI)	
Region of residence						
Attica	**0.63 (0.51–0.78)**	**0.76 (0.60–0.97)**	4.86	**0.72 (0.58–0.89)**	0.83 (0.66–1.05)	2.19
Aegean Islands and Crete	**0.67 (0.50–0.89)**	**0.66 (0.49–0.89)**	7.25	0.81 (0.61–1.07)	0.82 (0.61–1.09)	1.84
North Greece	1.21 (0.99–1.49)	1.20 (0.97–1.48)	3.03	1.06 (0.86–1.31)	1.07 (0.86–1.32)	0.39
Central Greece	1 (ref)	1 (ref)		1 (ref)	1 (ref)	
Degree of urbanization						
Urban areas	**0.78 (0.65–0.93)**	1.10 (0.89–1.36)	0.91	**0.77 (0.64–0.93)**	0.92 (0.75–1.14)	0.49
Rural areas	1 (ref)	1 (ref)		1 (ref)	1 (ref)	
Education level						
Primary education	**2.98 (2.36–3.76)**	**2.51 (1.89–3.34)**	40.60	**1.74 (1.39–2.18)**	**1.53 (1.16–2.02)**	9.14
Secondary education	**2.23 (1.82–2.73)**	**1.82 (1.47–2.27)**	29.69	**1.39 (1.15–1.68)**	1.19 (0.97–1.46)	2.78
Tertiary education	1 (ref)	1 (ref)		1 (ref)	1 (ref)	
Income status						
Lower income	**2.21 (1.83–2.67)**	**1.47 (1.18–1.83)**	11.96	**1.57 (1.30–1.90)**	1.17 (0.94–1.47)	2.10
Medium income	**1.64 (1.34–2.01)**	**1.27 (1.02–1.57)**	4.81	**1.46 (1.20–1.79)**	1.25 (1.02–1.55)	4.62
Higher income	1 (ref)	1 (ref)		1 (ref)	1 (ref)	
Employment status						
Employed	0.85 (0.71–1.01)	1.03 (0.83–1.29)	0.10	0.90 (0.76–1.07)	0.95 (0.77–1.19)	0.13
Unemployed	**1.59 (1.26–2.01)**	1.25 (0.92–1.71)	2.05	**1.48 (1.17–1.88)**	1.32 (0.96–1.81)	3.11
Retired	1 (ref)	1 (ref)		1 (ref)	1 (ref)	
Legal marital status						
Married	**0.59 (0.47–0.75)**	0.70 (0.48–1.03)	3.24	1.02 (0.79–1.32)	1.37 (0.93–2.03)	2.59
Never married	0.85 (0.66–1.09)	0.86 (0.63–1.17)	0.87	1.28 (0.97–1.68)	1.36 (0.99–1.88)	3.72
Widowed or divorced	1 (ref)	1 (ref)		1 (ref)	1 (ref)	
Household type						
Couple with children	**0.79 (0.65–0.96)**	1.01 (0.76–1.35)	0.01	0.90 (0.74–1.10)	0.84 (0.63–1.13)	1.24
Couple without any children	**0.64 (0.53–0.78)**	0.83 (0.59–1.17)	1.03	0.84 (0.69–1.02)	0.74 (0.52–1.04)	2.99
One‐person household or lone parent	1 (ref)	1 (ref)		1 (ref)	1 (ref)	

*Note:* The reference category for MD adherence is high adherence. Estimates with a significance level < 0.05 are highlighted in bold.

Abbreviation: MD, Mediterranean diet.

**Table 4:** Multinomial logistic regression analysis for MD adherence in females by considering all the socioeconomic and demographic variables alone (non‐adjusted ORs) or together (adjusted ORs).

	Low adherence vs. high adherence			Moderate adherence vs. high adherence		
	Non‐adjusted	Adjusted	Wald	Non‐adjusted	Adjusted	Wald
	OR (95% CI)	OR (95% CI)		OR (95% CI)	OR (95% CI)	
Region of residence						
Attica	**0.53 (0.43–0.65)**	**0.73 (0.58–0.92)**	6.95	**0.60 (0.49–0.74)**	**0.74 (0.59–0.92)**	6.89
Aegean Islands and Crete	**0.74 (0.56–0.99)**	0.77 (0.57–1.04)	2.87	1.00 (0.76–1.33)	1.03 (0.77–1.37)	0.05
North Greece	0.96 (0.77–1.18)	1.00 (0.80–1.24)	0.00	0.99 (0.80–1.22)	1.03 (0.82–1.28)	0.06
Central Greece	1 (ref)	1 (ref)		1 (ref)	1 (ref)	
Degree of urbanization						
Urban areas	**0.56 (0.46–0.68)**	0.91 (0.73–1.14)	0.91	**0.63 (0.52–0.76)**	0.88 (0.71–1.10)	1.14
Rural areas	1 (ref)	1 (ref)		1 (ref)	1 (ref)	
Education level						
Primary education	**4.44 (3.56–5.52)**	**3.13 (2.37–4.13)**	65.47	**2.23 (1.80–2.76)**	**1.73 (1.32–2.27)**	15.97
Secondary education	**2.13 (1.75–2.61)**	**1.83 (1.47–2.28)**	29.73	**1.51 (1.25–1.82)**	**1.31 (1.07–1.61)**	6.92
Tertiary education	1 (ref)	1 (ref)		1 (ref)	1 (ref)	
Income status						
Lower income	**2.76 (2.29–3.32)**	**1.76 (1.41–2.19)**	25.70	**1.75 (1.45–2.10)**	**1.32 (1.06–1.64)**	6.42
Medium income	**1.94 (1.59–2.36)**	**1.33 (1.07–1.65)**	6.79	**1.49 (1.23–1.81)**	1.19 (0.96–1.47)	2.73
Higher income	1 (ref)	1 (ref)		1 (ref)	1 (ref)	
Employment status						
Employed	**0.51 (0.42–0.62)**	0.94 (0.74–1.19)	0.23	**0.68 (0.56–0.83)**	0.87 (0.68–1.10)	1.35
Unemployed	0.89 (0.74–1.07)	0.94 (0.75–1.17)	0.26	1.98 (0.81–1.19)	0.94 (0.76–1.18)	0.21
Retired	1 (ref)	1 (ref)		1 (ref)	1 (ref)	
Legal marital status						
Married	**0.60 (0.51–0.72)**	0.86 (0.59–1.24)	0.63	0.93 (0.77–1.11)	1.21 (0.84–1.74)	1.04
Never married	**0.59 (0.48–0.74)**	0.88 (0.67–1.16)	0.70	0.85 (0.67–1.06)	1.08 (0.82–1.43)	0.36
Widowed or divorced	1 (ref)	1 (ref)		1 (ref)	1 (ref)	
Household type						
Couple with children	**0.67 (0.56–0.81)**	1.00 (0.72–1.38)	0.00	0.91 (0.76–1.10)	0.94 (0.68–1.30)	0.12
Couple without any children	**0.68 (0.56–0.82)**	0.81 (0.56–1.18)	1.14	0.95 (0.79–1.15)	0.82 (0.57–1.18)	1.10
One‐person household or lone parent	1 (ref)	1 (ref)		1 (ref)	1 (ref)	

*Note:* The reference category for MD adherence is high adherence. Estimates with a significance level < 0.05 are highlighted in bold.

Abbreviation: MD, Mediterranean diet.

Following a multivariant statistical analysis (discriminant analysis), it was revealed that the most important driver of MD adherence was education (Wilks' Lambda = 0.971; *p* < 0.001), followed by income (Wilks' Lambda = 0.976; *p* < 0.001), region of residence (Wilks' Lambda = 0.990; *p* < 0.001), sex (Wilks' Lambda = 0.991; *p* < 0.001), household type (Wilks' Lambda = 0.993; *p* < 0.001), employment status (Wilks' Lambda = 0.994; *p* < 0.001), urbanization (Wilks' Lambda = 0.995; *p* < 0.001) and age (Wilks' Lambda = 0.998; *p* < 0.001). Non‐significant association was observed for marital status (Wilks' Lambda = 1.000; *p* = 0.838). In particular, 33.69% of those in high MD adherence have tertiary education, compared to 23.86% in moderate MD adherence and 15.98% in low MD adherence. Similarly, 45.72% of high MD adherence group belong to the higher income group, compared to 34.46% of moderate MD adherence group and 27.72% of low MD adherence group. Additionally, 36.44% of people with high MD adherence reside in the Attica region, whereas the equivalent percentages for the moderate and low MD adherence categories are 27.96% and 25.95%, respectively. Fifty‐three percent (53%) of the high MD adherence group are males, while in low and moderate MD adherence groups, the percentage of males is 43.5%. Regarding the household type, 44.57% of those in low MD adherence live in one‐person households or as lone parents, compared to 38.13% and 34.81% in moderate and high MD adherence, respectively. Finally, 31.03% and 29.97% of those in low and moderate MD adherence, respectively, were unemployed, compared to 22.94% in high MD adherence. Especially for age and urbanization, although a statistically significant difference was observed, the range of difference is not considered significant demographically.

**Figure 2:**
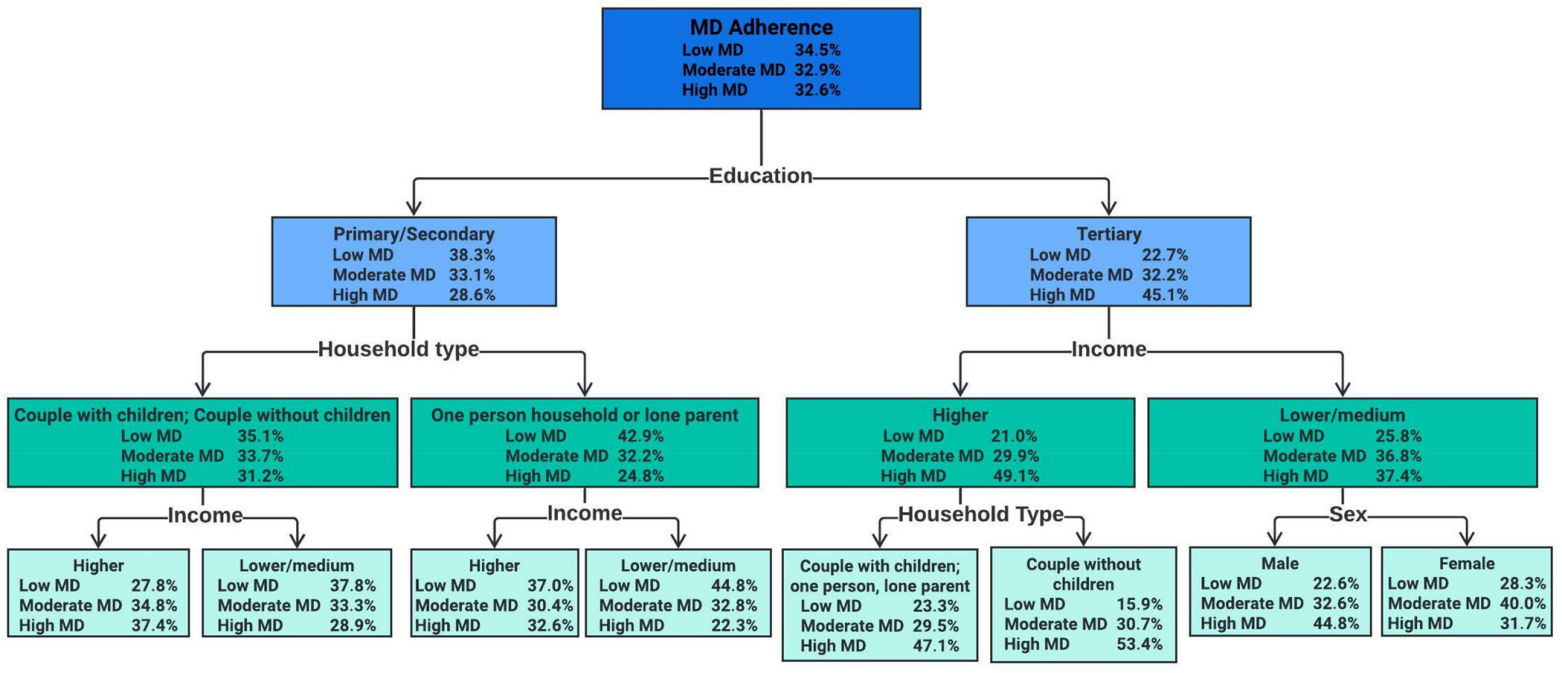
Decision tree obtained through CHAID method to predict the most significant paths of MD adherence groups.

Furthermore, decision tree analysis was applied (Figure 2) and revealed a number of paths that classified participants according to MD adherence, education, income, household type and sex. The most dominant classification factor was education, followed by income. For example, participants with tertiary education with higher income and living as couples without children were the group that most commonly adhered to high MD (53.4%). On the other hand, participants with primary/secondary education who live in one‐person households or are lone parents and belong to the lower and medium income groups are the most dominant in low MD adherence (44.8%).

Finally, regarding compliance with the national food‐based dietary guidelines, as shown in Table 5, although almost half of the population reported eating fruits and vegetables daily, only a very low percentage reported consuming the number of portions suggested by the guidelines. Moreover, less than one in five adhered to the recommendation of meat consumption, but the majority managed to consume the recommended weekly servings of legumes, poultry and fish. Education and income status seemed to be associated with compliance with dietary guidelines, as adherence rates for almost every guideline increased as the levels of education and income increased. The only exception to this finding is compliance with the recommendation for meat consumption (less than one serving per week), where individuals with primary education comply in a greater percentage than those with secondary or tertiary education.

**Table 5:** Percentage of participants that comply with specific national food‐based dietary guidelines, according to demographic and socioeconomic characteristics.

	Fruits	Fruits	Vegetables	Vegetables	Legumes	Red Meat	Poultry	Fish
	Daily	3 servings/day	Daily	4 servings/day	1–3 times/week or more	≤ 1 times/week	1–2 times/week	1–3 times/week
Total	48.5	8.7	51.3	3.4	81.3	18.6	81.3	60.2
Sex								
Males	**45.6**	8.9	**45.9**	3.7	80.9	**16.8**	81.0	60.8
Females	**51.0**	8.6	**56.0**	3.1	81.6	**20.2**	81.6	59.6
Region of residence								
Attica	**55.0**	**9.9**	**58.5**	**6.4**	**82.0**	**14.1**	**82.4**	**57.5**
Aegean Islands and Crete	**51.1**	**9.1**	**41.8**	**1.1**	**82.7**	**16.1**	**73.4**	**66.6**
North Greece	**49.3**	**10.2**	**51.6**	**2.4**	**78.4**	**20.3**	**81.7**	**59.9**
Central Greece	**39.1**	**5.5**	**46.5**	**2.2**	**83.3**	**22.7**	**82.9**	**61.0**
Degree of urbanization								
Urban areas	**49.9**	**9.2**	**53.4**	**3.9**	**80.4**	**17.5**	81.2	60.0
Rural areas	**44.3**	**7.3**	**44.6**	**2.1**	**84.0**	**22.1**	81.6	60.8
Education level								
Primary education	**44.2**	**7.2**	**40.5**	**1.6**	**83.5**	**25.3**	81.4	**58.2**
Secondary education	**45.0**	**7.8**	**50.0**	**3.4**	**79.3**	**16.1**	80.9	**58.6**
Tertiary education	**60.4**	**12.4**	**66.4**	**5.5**	**82.5**	**15.5**	81.9	**65.6**
Income status								
Lower income	**39.0**	**6.0**	**42.4**	**2.2**	81.1	19.9	80.0	**52.9**
Medium income	**49.4**	**9.0**	**51.5**	**2.8**	81.6	18.0	82.5	**61.9**
Higher income	**57.7**	**11.3**	**60.2**	**5.1**	81.2	17.7	81.8	**66.4**
Employment status								
Employed	**47.7**	9.1	**57.1**	**4.9**	**81.6**	**14.9**	82.2	**61.4**
Unemployed	**43.5**	8.0	**48.3**	**3.0**	**78.4**	**18.0**	79.9	**55.3**
Retired	**53.1**	9.0	**47.8**	**2.4**	**83.1**	**22.7**	81.5	**62.7**
Marital status								
Married or in a registered partnership	**51.9**	**9.5**	**55.2**	**3.7**	**85.6**	**16.2**	**82.6**	**64.8**
Never married	**43.4**	**7.5**	**49.5**	**4.3**	**74.2**	**16.6**	**79.2**	**53.3**
Widowed or divorced	**46.2**	**8.4**	**44.7**	**2.1**	**79.0**	**25.7**	**80.6**	**57.1**
Household type								
Couple with children	**48.6**	**8.9**	**55.9**	**4.7**	**83.8**	**11.8**	**83.6**	**62.8**
Couple without any children	**53.4**	**9.9**	**53.8**	**3.2**	**84.6**	**20.0**	**81.1**	**65.0**
One‐person household or lone parent	**44.6**	**7.7**	**45.7**	**2.7**	**76.8**	**22.7**	**79.7**	**54.4**

*Note:* Estimates with a significance level < 0.05 are highlighted in bold.

## 4 Discussion

The purpose of the current study was to examine the association between socioeconomic factors and MD adherence and to evaluate the socioeconomic profile of individuals with low adherence in comparison to the profiles of those who present moderate and high adherence in a representative sample of adults in Greece. Adherence of the Greek adult population to the MD pattern was low to moderate, and this was independent of age. These findings are in accordance with recent systematic reviews that showed moderate adherence to MD patterns both in Mediterranean countries and worldwide and in all age groups [12, 13].

According to sensitivity and cluster analysis, educational level emerged as the most significant factor associated with adherence to MD. Primary‐educated participants had nearly four times the probability of being in the lowest MD adherence tertile instead of the highest compared to tertiary‐educated participants. These findings are in accordance with previous results in Mediterranean and non‐Mediterranean countries [22, 24, 25], highlighting the impact of educational inequalities on health behaviour and the need to promote nutrition literacy for all population groups. Many nutrition education programmes have been offered to primary and secondary school students, and while the impact of these interventions has only been studied in a limited number of studies, some results show an improvement in students' adherence to healthy eating behaviours [35, 36, 37]. Nevertheless, nutrition education programmes designed for adults and older persons or for low‐educated groups are scarce. There is a need to include adult education interventions as well as nutrition literacy programmes for vulnerable groups in future nutrition policies.

Another finding of the current study is that people with a lower income have higher odds of having low MD adherence compared to persons with a higher income. Income is also associated with adherence to recommendations for specific food groups. Family income is a significant contributor to food choices and low financial status is linked to low adherence to healthy eating patterns [22, 24, 25, 28, 38, 39]. This should be addressed in conjunction with the fact that many products compatible with the MD pattern (such as fruits, olive oil and fish) have become expensive in recent decades, thus making MD less affordable [18, 20]. Providing reduced tax rates and subsidies for specific foods (such as fruits, vegetables, legumes, fish and olive oil) could be an effective policy to improve the dietary habits of low‐income groups [40, 41]. The World Health Organization recommends the use of food subsidies not only to improve affordability and increase the consumption of healthy foods but also to achieve the right to health [42].

The current study has also shown that participants with low adherence to MD are more likely to be unemployed, widowed or divorced and to live in one‐person households. Considering these factors, previous studies yielded ambiguous results. Employed participants had higher MD adherence scores in a study in Australia [25], whereas studies in Portugal and Italy did not find significant differences between employed and unemployed participants [24, 43]. Regarding marital status, a recent study in Cyprus found similar results to the current study, as being divorced or widowed was negatively associated with the MD score [28]. Marital status has been identified as a factor that influences eating habits, with persons who are married or living with a partner eating healthier than those who are single, divorced or widowed [25, 44, 45]. This finding may be explained by the fact that a couple living together will probably have more money overall to spend on food and by the fact that they are more likely to prefer healthier options because they are caring for children. An interesting result of the current study is that the cluster with the higher MD adherence was the one with high education, high income and living as couples without children. This could be explained by the fact that couples without children can spend more money on healthy food options compared to families with children. On the other hand, individuals who live alone might choose to order takeout, eat out of home or prefer low‐cost meal options. As it was indicated, one‐person households with low income and low education had the higher odds of low MD adherence. However, more research is necessary because aspects, including marital status, household type and occupation, have not been fully examined in relation to MD adherence.

Finally, it is worth discussing that the current study found that significantly more females than males had low MD adherence. However, sex differences in MD adherence disappeared when alcohol consumption was excluded from the total MD score. Women reported lower consumption of alcohol, which resulted in a score of zero for this MD factor, while they had greater scores for all other factors. As confirmed in the current study, women had greater compliance with the specific food group consumption guidelines than men. Many studies have indicated no difference in MD adherence between sex groups, whereas other studies have found greater MD adherence among women [46]. The important role of women in transmitting knowledge of the MD is highlighted in the Decision of UNESCO for the inscription of the MD on the List of the Intangible Cultural Heritage of Humanity [47]. Supporting women in being educated regarding healthy eating, cooking and feeding of family members could be an effective way to promote MD adherence. Furthermore, sex stratification in nutritional studies needs to be improved, as there is a lack of female representation and understanding of sex‐specific interactions [48].

## 5 Limitations and Strengths

The current study has some limitations that should be considered when drawing conclusions. First, owing to the study's cross‐sectional methodology, no causal associations could be identified. Second, concerns regarding recall bias may have arisen. This could have happened because the interviewers were adequately trained, but they were not health professionals, and they may have been unable to explain some details regarding food consumption questions to the participants. Nevertheless, they provided detailed written explanations and photographs of food types and quantities. Another limitation is that the effects of other factors not assessed in this study cannot be entirely excluded. For example, there are no data regarding the different subregions. Attica is a large region with many different levels of socioeconomic status; thus, an analysis of sub‐region‐specific socioeconomic characteristics would be useful. Finally, it was not possible to derive more detailed nutrition data, such as daily energy and macronutrient intake and consumption of fast food.

Although these findings should be interpreted with caution, the current study has several strengths. This is the first secondary analysis of data on food consumption by the national section of the EHIS. The study population is representative of the Greek adult population in terms of sex, age and geographical distribution, while the sample size was relatively large compared to previous studies of the Greek population. Moreover, the EHIS collects data on a significant number of demographic and socioeconomic factors. Finally, MD adherence was evaluated using MedDietScore, a validated questionnaire specifically designed to collect information on adherence to MD.

## 6 Conclusion and Future Perspectives

The health benefits of MD have been established since the Seven Countries Study by Ancel Keys, and the Greek government occasionally launched initiatives to encourage the adoption of this traditional eating pattern. However, the majority of people in this Mediterranean country do not follow MD, as shown by both the results of this survey and others. Socioeconomic factors, primarily income and education status, are the main contributors to the low adherence levels that have been reported during the last decades.

Nevertheless, socioeconomic inequalities are not considered by public authorities when designing policies to enhance healthy eating habits among the population. Specific actions addressing socioeconomic inequalities should make healthy food items more accessible and affordable to make MD appealing to all parts of the community. Some examples include lowering the cost of items compatible with the MD pattern, either through food subsidies or decreased taxes, and providing meals through food aid programmes tailored to the MD pattern. Furthermore, ongoing programmes to improve nutrition literacy should target people of all ages and socioeconomic statuses, while local community authorities should be able to identify and assist vulnerable individuals and families in following healthy eating patterns.

Results from longitudinal studies on MD adherence will provide essential information to public authorities to evaluate and improve future policy actions. The EHIS could be an important source of information, as it is conducted every 5 years under a common methodology in all EU countries. For the first time in 2019, Greece was able to collect data regarding all food groups, not only fruits and vegetables; thus, it was possible to evaluate the adherence to an eating pattern, not only in specific food groups but in a country‐representative sample. Integrating these questions into other nations' EHIS questionnaires would allow for future intercountry comparisons, as the harmonized EHIS data are highly comparable. It will also be possible to perform longitudinal comparisons between 2019 and future EHIS data.

## Author Contributions

The authors contributed to the following aspects of research: **Ioanna Kontele:** data processing and analysis, writing – original draft. **Demosthenes Panagiotakos:** data analysis, interpretation of data, review and editing of the manuscript. **Mary Yannakoulia:** interpretation of data, review and editing of the manuscript. **Tonia Vassilakou:** supervision, interpretation of data, review and editing of the manuscript.

## Ethics Statement

This project did not involve human subjects research as it relied on secondary analysis of de‐identified data. Therefore, ethics approval was not required. Access to anonymized microdata was obtained upon request from the Statistical Data Dissemination Section of the Statistical Information and Publications Division of the Hellenic Statistical Authority (reference number ΓΠ‐649/23‐09‐2022). The survey was conducted pursuant to Regulation (EC) No. 1338/2008 of the European Parliament and Council, laying down issues concerning community statistics on public health and occupational health and safety, and pursuant to Implementing Regulation (EC) No 255/2018, laying down the basic concepts and variables included in the survey questionnaire.

## Conflicts of Interest

The authors declare no conflicts of interest.

### Transparent Peer Review

The peer review history for this article is available at https://www.webofscience.com/api/gateway/wos/peer-review/10.1111/jhn.70023.

## Transparency Declaration

The lead author affirms that this manuscript is an honest, accurate and transparent account of the study being reported. The reporting of this work is compliant with STROBE guidelines. The lead author affirms that no important aspects of the study have been omitted and that any discrepancies from the study as planned have been explained.

## Data Availability

Data that support the findings of this study are available from the Hellenic Statistical Authority. Restrictions apply to the availability of these data, which were used under license for this study. Data are available from the author(s) with the permission of the Hellenic Statistical Authority.
